# Utility of the pooling approach as applied to whole genome association scans with high-density Affymetrix microarrays

**DOI:** 10.1186/1756-0500-3-274

**Published:** 2010-11-01

**Authors:** Alexandra Schosser, Katrina Pirlo, Darya Gaysina, Sarah Cohen-Woods, Leonard C Schalkwyk, Amanda Elkin, Ania Korszun, Cerisse Gunasinghe, Joanna Gray, Lisa Jones, Emma Meaburn, Anne E Farmer, Ian W Craig, Peter McGuffin

**Affiliations:** 1 MRC SGDP Centre, Institute of Psychiatry, King's College London, London, UK; 2 Centre for Psychiatry, Wolfson Institute of Preventive Medicine, Barts and The London Medical School, Queen Mary University of London, London, UK; 3 Department of Psychiatry, University of Birmingham, Birmingham, UK; 4 Division of Biological Psychiatry, Department of Psychiatry and Psychotherapy, Medical University Vienna, Vienna, Austria

## Abstract

**Background:**

We report an attempt to extend the previously successful approach of combining SNP (single nucleotide polymorphism) microarrays and DNA pooling (SNP-MaP) employing high-density microarrays. Whereas earlier studies employed a range of Affymetrix SNP microarrays comprising from 10 K to 500 K SNPs, this most recent investigation used the 6.0 chip which displays 906,600 SNP probes and 946,000 probes for the interrogation of CNVs (copy number variations). The genotyping assay using the Affymetrix SNP 6.0 array is highly demanding on sample quality due to the small feature size, low redundancy, and lack of mismatch probes.

**Findings:**

In the first study published so far using this microarray on pooled DNA, we found that pooled cheek swab DNA could not accurately predict real allele frequencies of the samples that comprised the pools. In contrast, the allele frequency estimates using blood DNA pools were reasonable, although inferior compared to those obtained with previously employed Affymetrix microarrays. However, it might be possible to improve performance by developing improved analysis methods.

**Conclusions:**

Despite the decreasing costs of genome-wide individual genotyping, the pooling approach may have applications in very large-scale case-control association studies. In such cases, our study suggests that high-quality DNA preparations and lower density platforms should be preferred.

## Background

We report an attempt to extend the previously successful approach of SNP (single nucleotide polymorphism) microarrays and DNA pooling (SNP-MaP) [1-14]. Whereas earlier studies had employed a range of Affymetrix SNP microarrays interrogating between 10 K to 500 K SNPs [15], we used the Affymetrix SNP 6.0 which displays 906,600 SNP probes and 946,000 probes for the interrogation of CNVs (copy number variations). We have performed this genome-wide association study (GWAS) using pooled DNA from a large depression case-control sample (1418 cases, 1301 controls), and the SNPs with the largest differences between cases and controls were individually genotyped in the sample used to construct the pools. In contrast to the suitability of Affymetrix microarrays (up to the 500 K) for successful analysis of DNA pools as established by other groups, the properties of the Affymetrix SNP 6.0 used in the current study were unproven at the time of investigation.

## Methods

### Study design

GWAS using phenotypically standardised pooled DNA of a large depression case-control (DeCC) sample was followed-up by individually genotyping the 'top-hit' SNPs. The SNP-MaP approach was conducted with the Affymetrix Genome-Wide Human SNP Array 6.0, containing approximately 1 million SNP markers [1].

### Samples

Our depression case-control (DeCC) samples of 1418 patients with a diagnosis of recurrent major depression (MDD) and 1301 control subjects were recruited from three clinical sites in UK (London, Cardiff and Birmingham) as described previously [16]. All participants gave written informed consent, and the study was approved by the Local Ethical Committees of the three centres.

### DNA pooling

Blood samples were obtained from all patients and either blood or buccal mucosa swabs obtained from controls. Genomic DNA was extracted by an in-house validated procedure as described previously [17,18]. Genomic DNA was quantified three times using PicoGreen^® ^assay (Molecular Probes, Eugene, OR, USA). The DNA pools of the DeCC sample were created as follows: first of all, the sample was divided into cases and controls, and then into males and females. Subsequently, these groups were divided according to their body mass index (BMI <25, BMI 25-30, BMI >30) in the light of phenotypic analyses of DeCC, showing strong associations between depression and various physical diseases mediated via increased BMI [19]. We randomly created 57 DNA-pools of on average 47.67 individuals of matched sex and phenotype (SD = 9.39), and each individual contributed 100 ng DNA to their pools which ranged in concentrations from 19.0 to 24.99 ng/μL.

### Allelotyping the DNA pools

The Affymetrix Genome-Wide Human SNP Array 6.0 in combination with the standard Affymetrix protocol was used to allelotype 200 ng genomic DNA from each pool. Water was used as a negative control to test for contamination, and to test for assay performance, the manufacturer's individual reference DNA was used as a positive control. Three pools were genotyped in duplicate, serving as technical replicates.

### Generating RAS (relative allele signal) scores

In contrast to the earlier versions of Affymetrix genotyping arrays, the latest SNP arrays (Affymetrix Genome-Wide SNP Array 5.0 and 6.0; but not the previously employed 500 K chip) differ in that the mismatch probes have been discarded in favour of greater perfect match probe density. Thus, mismatch intensities can no longer be subtracted before calculating RAS scores, and RAS scores based on an equivalent principle to those that have been validated for pooling in previous work cannot, therefore, be calculated anymore. As a consequence they may no longer represent a good estimate of the absolute allele frequency in the pool; however, such estimates may still be useful for detecting frequency differences between pools [20].

### Confirmatory individual genotyping

Confirmatory individual genotyping was performed using the Sequenom MassARRAY^® ^iPLEX Gold assay and TaqMan^® ^SNP genotyping platform.

### Statistical analyses

Cell intensity (.CEL) files were exported and RAS scores generated from the cell intensity data using the RAS score algorithm as implemented in a freely available script in R (http://sgdp.iop.kcl.ac.uk/oleo/affy), which has now become part of an R package, the SNPMaP package [21]. To test for significant differences between cases and controls, an independent Student's t-Test was applied using R statistical software (http://www.r-project.org/).

## Results

**Figure 1:**
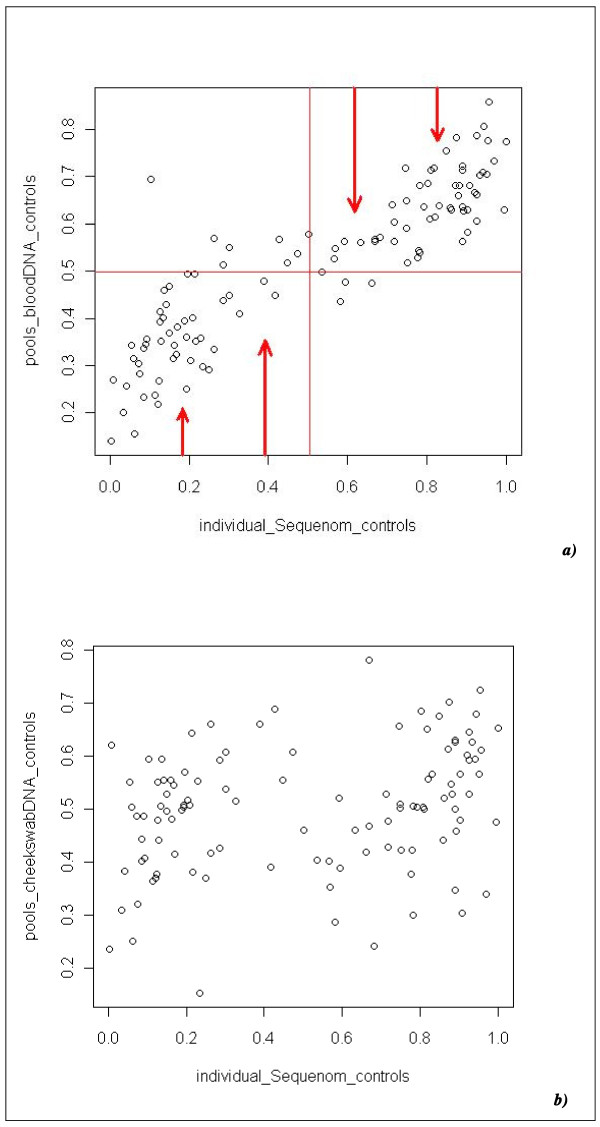
**Pearson's correlation of real allele frequencies (x-axis) and frequency estimates from pooled DNA (RAS-scores, y-axis) of BACCS controls *(a) *(blood DNA) and the DeCC controls *(b) *(cheek swab DNA) are shown**. The red arrows ***(a) ***indicate, that the distribution of RAS scores (y-axis) are now slumped towards 0.5 as a consequence of the discarded mismatch probe (thus mismatch probe intensities cannot be substracted from perfect match probe intensities) as compared to previous Affymetrix microarrays up to the 500 K. Pooled cheek swab DNA **(*b*) **could not sufficiently predict 'real' allele frequencies of the samples that comprised the pools.

We have performed a genome-wide association study (GWAS) using pooled DNA of a depression case-control sample and the Affymetrix Genome-Wide Human SNP Array 6.0, and followed-up the 'top-hit' SNPs using confirmatory individual genotyping with Sequenom MassARRAY^® ^iPLEX Gold or TaqMan^®^. Three DNA-pools were genotyped in duplicate and hybridized to two separate microarrays ('technical replicates'). The pairwise correlations were 0.945, 0.965 and 0.967 respectively. Biological replicates (pools of the same phenotypic group) were available for the majority of pools in the current study. We randomly selected five blood DNA case-pools of the same phenotypic group (cases, females, body-mass-index 25 to 30) and found an average pairwise correlation of 0.956; we found similar average pairwise correlations (0.959) for four cheek-swab-DNA control pools of the same phenotypic group (controls, females, body-mass-index less than 25) and four blood DNA control-pools of the same phenotypic group (0.967) (controls, females, body-mass-index less than 25). We allelotyped a total of 57 DNA pools of on average 47.67 individuals each (SD = 9.39), 29 pools of cases and 28 pools of controls. All case-pools contained blood DNA, whereas only 10 control-pools contained blood DNA, the remainder (18) contained cheek swab DNA. To test for significant differences in allele frequencies between cases and controls, an independent Student's t-Test was applied and after excluding rare variants with a minor allele frequency (MAF) <0.05, we found 74 SNPs crossing the genome-wide significance threshold of 7.2 × 10^-8 ^[22]. The top-ranked SNPs of the pooling GWAS were followed-up by individually genotyping the samples used to construct the pools, and the validity of the SNP-MaP approach was assessed by comparing allele frequency estimates from pooled DNA (RAS scores) with individual genotyping data of 110 SNPs genotyped with either Sequenom MassARRAY^® ^iPLEX Gold (108 SNPs) or TaqMan^® ^(2 SNPs). We performed these analyses for cases and controls separately and, since all case pools contained blood DNA but 18 out of 28 control pools contained cheek swab DNA, we divided the controls into two groups according to source of DNA. The Pearson's correlation of the allele frequency estimates of the DNA pools (RAS scores) and the 'real' allele frequencies derived from individual genotyping was 0.9010 for the case-pools (all blood DNA) and 0.8853 for the blood DNA control-pools; however, the correlation was only 0.2734 for the cheek swab control-pools (see Figure 1).

## Conclusions

The current study was an attempt to extend the previously successful approach of SNP microarrays and DNA pooling (SNP-MaP) to a whole-genome association study of MDD employing high-density microarrays. Whereas the earlier studies had employed a range of Affymetrix SNP microarrays comprising from 10 K to 500 K SNPs, this most recent investigation used the 6.0 chip, which displays 906,600 SNP probes and 946,000 probes for the interrogation of CNVs. The main advantage of DNA pooling is that it is a way to reduce costs in determining allele frequencies in large case/control cohorts. Since individual genotypes cannot be determined, this process is called "allelotyping" and it enables allele frequency differences between pools comprised DNA from many individuals to be compared without the need to genotype each individual.

For the purposes of allelotyping pooled DNA, there are three subtle but potentially detrimental changes differentiating the Affymetrix SNP 6.0 microarray from its validated 10 K, 100 K and 500 K predecessors. First, the feature size has decreased from 18 μm on the 10 K, to 8 μm on the 100 K, and to 5 μm on the 500 K and Affymetrix SNP 6.0 microarrays. Second, each SNP is interrogated by 40 probes on the Affymetrix 10 K and 100 K, 24 probes for 90% of the Affymetrix 500 K, but by only 6-8 probes on the Affymetrix SNP 6.0 microarray. Third, the mismatch probes have been discarded from the Affymetrix SNP 6.0 microarray, and thus mismatch probe intensities cannot be subtracted from perfect match probe intensities as it was the case for previous Affymetrix microarrays up to the 500 K. To our knowledge, this is the first study published so far using the Affymetrix SNP 6.0 microarray on pooled DNA.

We created 57 DNA pools of the DeCC sample, each containing equal amounts of DNA of on average 47.67 individuals (SD = 9.39).

We assessed validity of the genome-wide pooling study by comparing the estimates of allele frequencies from pooled DNA with individual genotyping data of 110 SNPs genotyped with either Sequenom MassARRAY^® ^iPLEX Gold or TaqMan^®^, and allele frequency estimates provided by Affymetrix NetAffx™ (http://www.affymetrix.com/analysis/index.affx). While there was a high correlation (>0.99) between allele frequencies derived from confirmatory individual genotyping and the allele frequencies provided by Affymetrix NetAffx™, the correlation between the allele frequency estimates derived from the pools (RAS scores) and the 'real' allele frequencies derived from individually genotyping the samples which composed the pools was somewhat disappointing. Although the correlation of RAS scores and 'real' allele frequencies was reasonable (but rather poor as compared to the previous Affymetrix microarrays up to the 500 K) for the blood DNA cases (Pearson's correlation of 0.901) and the blood DNA controls (Pearson's correlation of 0.885), the correlation of cheek swab DNA controls was only 0.273. The RAS scores produced using the Affymetrix SNP 6.0 array have a different distribution than the conventional RAS scores that had been validated for pooling in previous work, since the mismatch probes have been discarded in favour of greater perfect match probe intensity. Therefore, mismatch intensities cannot be substracted before calculating RAS scores, and the ratio of allele signal intensities A/(A+B) is calculated instead. As a consequence, the RAS scores derived from the Affymetrix SNP 6.0 array are not necessarily a good estimate of the absolute allele frequency in the pool [21]. However, this should not affect case-control comparison in a genome-wide pooling study, since we are interested in frequency differences between the groups, not necessarily in absolute allele frequencies. Although the RAS scores indeed did not accurately predict the 'real' allele frequencies in our sample, we hypothesized that the difference between cases and controls are expected to be the same in the pools as in the individuals that comprise the pools. To test this hypothesis, we calculated the ratio of the mean allele frequencies of controls and cases (controls/cases) for the 110 SNPs individually genotyped, and the ratio of the mean RAS scores of control and case pools (control pools/case pools) for the same SNPs genotyped on the Affymetrix SNP 6.0 array. Subsequently, we calculated the ratio of the two ratios, where a ratio of 1 would indicate that the mean control/case ratio of the RAS scores for a given SNP was a perfect estimate of the 'real' ratio derived from individual genotyping of the samples composing the pools. We found that the mean of the latter ratios was 1.01 and the standard deviation (SD) was 0.25, with 74.55% of SNPs within one SD. Performing the same analyses after separating blood and cheek swab controls, we found a mean of 0.99 and SD of 0.11 in case of blood controls (89.09% of SNPs within 1 SD), and a mean of 1.05 and SD of 0.39 in case of cheek swab controls (74% of SNPs within 1 SD).

These findings suggests that the microarray allelotyping cheek swab DNA pools did not sufficiently predict 'real' allele frequencies of the samples comprising the pools (see Figure 1) even though the quality of cheek swab DNA employed was comparable to that in previous studies. The genotyping assay using the Affymetrix SNP 6.0 array is highly demanding on sample quality due to the small feature size, low redundancy, and lack of mismatch probes. In contrast to the problems encountered with cheek swab DNA, the allele frequency estimates using blood DNA pools on the 6.0 chip were reasonable, although inferior compared to those obtained with previous Affymetrix microarrays up to the 500 K chip. Nevertheless, knowing the nature of the problems encountered and the differences in the organisation of the probe features distinguishing the Affymetrix SNP 6.0 array from its predecessors, it might be possible, however, to improve performance by developing improved analysis methods. In addition, despite the decreasing costs of genome-wide individual genotyping, the pooling approach with this (and other arrays) may still be applicable in very large-scale case-control association studies, preferably using high-quality blood DNA or a less SNP dense platform as appropriate.

## Competing interests

The authors declare that they have no competing interests.

## Authors' contributions

AS carried out the molecular genetic studies, the statistical analyses and drafted the manuscript; KP performed pool construction and quantification; DG participated in quantification and genotyping. SCW participated in pool construction; LS supervised statistical analyses; AE, A, CG, JG and LJ participated in sample collection; EM supervised microarray genotyping; AF, IC and PM were principal investigators of the proposed study. All authors read and approved the final manuscript.

## Note added in proof

Since the revised, peer reviewed version of this paper was submitted, another study using the Affymetrix 6.0 on pooled DNA has been published (Chiang CWK, Gajdos ZKZ, Korn JM, Kuruvilla FG, Butler JL, et al. (2010) Rapid Assessment of Genetic Ancestry in Populations of Unknown Origin by Genome-Wide Genotyping of Pooled Samples. PLoS Genet 6(3): e1000866. doi:10.1371/journal.pgen.1000866.). This study shows that genotyping pooled DNA is a valid approach for identifying SNPs with large allele frequency differences and does not contradict the findings presented here.
